# Interpreting heart rate variability in clinical context: a framework for understanding a multidimensional autonomic biomarker

**DOI:** 10.3389/fcvm.2026.1949249

**Published:** 2026-09-14

**Authors:** Bahjat Z. Ghazzal, Ibrahim Hasan, Nebras Hasan, Ziyad Ghazzal, Marwan M. Refaat

**Affiliations:** 1 Department of Medicine, University of Massachusetts Chan Medical School, Worcester, MA, United States; 2 Department of Internal Medicine, Division of Cardiology, American University of Beirut Medical Center, Beirut, Lebanon

**Keywords:** autonomic regulation, cardiovascular risk assessment, context-aware interpretation, heart rate variability, nonlinear HRV

## Abstract

Heart rate variability (HRV) is a non-invasive marker of autonomic cardiovascular regulation that reflects beat-to-beat fluctuations in cardiac cycle length. Reduced HRV has been associated with adverse cardiovascular outcomes across multiple disease states, yet its clinical use remains limited by substantial interpretive complexity. Unlike static biomarkers, HRV is highly sensitive to measurement modality, recording duration, circadian timing, behavioral factors, environmental exposures, medications, and comorbidities. Therefore, isolated HRV values cannot be interpreted as direct indicators of autonomic health without careful consideration of context. This review summarizes the physiological basis of HRV, key time-domain, frequency-domain, and nonlinear/complexity metrics, and major acquisition methods, including 24 h electrocardiography (ECG), short-term ECG, chest strap monitors, and wrist-worn devices. It also examines how sleep, physical activity, diet, psychological stress, substance use, age and sex, medications, and cardiovascular or metabolic diseases influence HRV interpretation. We propose a context-aware framework for interpreting HRV as a dynamic physiological signal rather than a standalone diagnostic marker. When appropriately contextualized, HRV may serve as a complementary tool for cardiovascular risk assessment and provide insight into residual risk not fully captured by conventional clinical models.

## Introduction

Cardiovascular medicine has made substantial progress in risk stratification through the identification of increasingly precise biomarkers and clinical risk models. Yet despite advances across diagnostics, therapeutics, and prevention strategies, cardiovascular disease remains the leading global cause of death, and a significant burden of cardiovascular events persists among individuals who appear optimally treated by conventional metrics (1). This phenomenon, often described as *residual cardiovascular risk*, suggests that important biological pathways influencing cardiovascular outcomes remain incompletely captured by existing tools (2, 3).

One such pathway may lie in the autonomic regulation of the cardiovascular system. Heart rate variability (HRV), a measure of beat-to-beat fluctuations in cardiac cycle length, reflects the dynamic interaction between sympathetic and parasympathetic influences on the heart (4). For decades, reduced HRV has been associated with adverse cardiovascular outcomes. Despite this extensive literature, HRV has remained largely confined to research settings and has not been broadly adopted into routine clinical risk assessment. However, the increasing availability of wearable technologies capable of continuously estimating HRV has renewed interest in its clinical relevance.

As HRV moves from controlled research environments into everyday clinical and consumer settings, its sensitivity to context becomes not a theoretical concern, but a central challenge to safe interpretation. Unlike traditional biomarkers that provide static snapshots of physiology, HRV represents a dynamic, non-invasive signal that integrates neural, metabolic, inflammatory, behavioral, and environmental influences over time (5). This breadth is arguably both HRV's greatest strength and its greatest limitation. In contrast to traditional biomarkers such as low-density lipoprotein cholesterol or cardiac troponin, HRV is highly sensitive to context. Several factors including but not limited to measurement conditions, sleep, physical activity, psychological stress, substance use, existing disease states, and medications can all meaningfully alter HRV independent of underlying cardiovascular pathology. Failure to account for this context has contributed to substantial heterogeneity and apparent inconsistency across HRV studies. Associations between HRV and cardiovascular outcomes often vary by study design, recording duration, analytic method, population characteristics, and behavioral environment at the time of measurement. Rather than reflecting a fundamental weakness of HRV as a biomarker, this discordance likely reflects inappropriate or incomplete interpretation of a signal that is inherently dynamic and context dependent.

To date, most reviews of HRV in cardiovascular disease have focused on summarizing prognostic associations or describing analytic techniques. Far fewer have addressed the central interpretive challenge posed by HRV, namely, how to distinguish meaningful autonomic dysfunction from benign or reversible variability driven by context. There remains no recent nor widely adopted framework to guide clinicians and researchers in safely and consistently interpreting HRV across different measurement modalities, environments, and clinical populations.

In this review, we propose a practical interpretive framework for HRV that explicitly incorporates measurement context, timing, behavioral and environmental influences, underlying disease states and corresponding medication use. We review the physiological basis of HRV, summarize key measurement approaches and metrics, and examine how lifestyle factors and disease states modify HRV signals. Rather than positioning HRV as a replacement for established cardiovascular biomarkers, we argue that HRV may serve as a complementary tool for capturing dimensions of residual cardiovascular risk that are not reflected in traditional models, provided it is interpreted with appropriate caution. By formalizing a context-aware approach to HRV interpretation, this review aims to support more consistent research findings and safer clinical application as HRV data become increasingly accessible in modern cardiovascular care.

## Physiological basis of heart rate variability

HRV reflects the beat-to-beat fluctuations in cardiac cycle length between successive heartbeats. These fluctuations arise from the dynamic interplay between parasympathetic and sympathetic inputs of the autonomic nervous system (ANS), which continuously modulate heart rate in response to physiological demands (4).

Under resting conditions, parasympathetic (vagal) tone typically predominates, slowing heart rate via vagal input to the sinoatrial and atrioventricular nodes (6). In contrast, sympathetic activation increases heart rate through β_1_-adrenergic signaling, enhancing nodal conduction and myocardial contractility (6). As such, these systems operate on distinct temporal scales. Vagal effects are rapid and can influence heart rate on a beat-to-beat basis, whereas sympathetic responses develop more slowly and are generally more sustained (7, 8). As a result, changes in heart rate between successive beats are primarily mediated by parasympathetic activation or withdrawal (9). In addition to central autonomic inputs, cardiac control is shaped by an intrinsic cardiac nervous system composed of interconnected intracardiac ganglia and local neural circuits (10, 11). This intrinsic system integrates extrinsic autonomic signals with local reflexes, enabling fine, beat-to-beat adjustment of heart rate and conduction.

The physiological consequence of this organization is that HRV reflects autonomic flexibility and responsiveness rather than a fixed property of cardiovascular health. Reduced HRV may indicate impaired autonomic regulation due to structural or systemic disease, but it may also represent an adaptive response to acute physiological or environmental stress. Identical HRV values can therefore arise from fundamentally different underlying states, depending on context and biological substrate. This property distinguishes HRV from many established cardiovascular biomarkers. Traditional markers are often less sensitive to short-term physiological, behavioral, or environmental variation, but they typically reflect downstream manifestations of disease processes that are already established. In contrast, HRV may be able to capture more upstream autonomic strain and physiological stress that precede overt structural pathology. This dual nature makes HRV both more challenging to interpret and potentially more informative when considered alongside conventional biomarkers. Understanding this distinction is essential before applying HRV to risk stratification and motivates the need for a structured interpretive framework developed in the sections that follow.

## Heart rate variability metrics: interpretive implications of metric selection

HRV is not a single physiological quantity, but a family of related metrics derived from the same underlying R–R interval signal. Although all HRV metrics quantify variability in cardiac cycle length, they differ in how that variability is summarized and over what temporal window it is captured. As a result, individual metrics emphasize distinct aspects of autonomic regulation and are best understood as complementary indices rather than interchangeable measures. Careful attention to metric selection is therefore essential for accurate interpretation of HRV findings and their implications for cardiovascular risk.

In this section, we review the most commonly used HRV metrics across three major categories. Time-domain parameters include the standard deviation of all normal-to-normal intervals (SDNN), the root mean square of successive differences (RMSSD), and the percentage of adjacent intervals differing by more than 50 ms (pNN50). Frequency-domain parameters include high-frequency (HF) power, low-frequency (LF) power, and the LF:HF ratio, which remain the most frequently reported spectral measures in cardiovascular literature. Nonlinear and complexity-domain parameters, including Poincaré-plot indices, detrended fluctuation analysis, and entropy-based measures, characterize the temporal organization and regularity of R–R interval dynamics rather than the amount of variability present. Detailed technical treatments of these metric families and of their measurement in clinical settings are available elsewhere and remain the appropriate reference for questions of measurement technique (12); our concern here is with the interpretive consequences of metric selection rather than with the mechanics of computation.

### Time-domain parameters

Time-domain measures are the most direct descriptors of HRV, calculated from intervals between successive normal heartbeats over a defined recording period. These metrics quantify the overall amount of variability present, without distinguishing the speed or frequency at which fluctuations occur, and are generally reliable across both short- and long-term recordings (13). Commonly used time-domain parameters include the standard deviation of all normal-to-normal intervals (SDNN), the root mean square of successive differences (RMSSD), and the percentage of adjacent intervals differing by more than 50 ms (pNN50).

SDNN is the most comprehensive time-domain HRV metric and, when derived from 24 h recordings, is widely regarded as a gold-standard index of overall HRV (4). Over extended recordings, SDNN integrates variability arising from multiple regulatory processes operating across different time scales, including short-term fluctuations related to respiration, activity, and stress, as well as slower variability driven by circadian rhythms and behavioral transitions (4). As a global measure, however, SDNN carries important interpretive limitations. It does not distinguish between parasympathetic and sympathetic contributions to HRV, such that similar SDNN values may reflect different underlying autonomic states. For this reason, SDNN is most informative when interpreted alongside other HRV metrics that provide greater physiological specificity. In addition, SDNN exhibits predictable circadian variation, with higher values typically observed during nighttime rest and lower values during daytime activity, reflecting normal shifts in autonomic balance (14, 15). Failure to account for these temporal patterns may lead to misinterpretation of autonomic health, particularly when comparing measurements obtained at different times of day. Despite these limitations, SDNN demonstrates relatively high agreement between certain smartwatch-derived estimates and electrocardiographic measurements, whereas other HRV metrics, including RMSSD and high-frequency components, may show only moderate concordance (16). Consequently, SDNN is commonly favored for HRV assessment in wearable-based and ambulatory monitoring contexts.

RMSSD quantifies beat-to-beat variability in heart rate and primarily reflects short-term parasympathetic modulation (4). Because RMSSD is calculated from successive R–R interval differences, it captures rapid fluctuations in heart rate and is therefore well suited to short recording durations. Standard 5 min recordings provide reliable estimates of RMSSD, and studies have shown that recordings as brief as 1–2 min, and in some contexts even 30 s, correlate reasonably well with 5 min values under resting conditions (17, 18). In contrast to SDNN, RMSSD is relatively insensitive to recording length once a minimum duration is met, as it does not incorporate slower oscillatory processes (19). RMSSD correlates strongly with other vagally mediated HRV metrics, including pNN50 and HF power (20, 21). Because these measures share overlapping physiological significance, RMSSD is commonly favored in contemporary HRV analyses due to its numerical stability, ease of interpretation as an absolute value, and feasibility across a wide range of recording durations.

pNN50 quantifies the percentage of successive normal R–R intervals differing by more than 50 ms (4). Like RMSSD, pNN50 is heavily influenced by high-frequency variations and therefore is considered a marker of parasympathetic activity (22, 23). Its sensitivity to short-term autonomic fluctuations makes pNN50 a practical choice for monitoring dynamic changes in vagal tone, particularly in contexts where quick adaptability of the ANS is crucial (24). A higher pNN50 indicates that a large proportion of consecutive heart beats have substantially different cycle lengths, implying a dynamically responsive vagal modulation of the heart rate. The 50 ms cutoff used in pNN50 represents one convention within a broader family of pNNx metrics defined by different thresholds. It has been proposed that lower thresholds (e.g., 20–40 ms) may better capture physiologically meaningful beat-to-beat variability, particularly in individuals with low overall HRV (23). As a result, reliance on a fixed 50 ms threshold may reduce sensitivity in low-HRV states and limit the interpretive advantage of pNN50 relative to other parasympathetic indices.

Together, time-domain HRV metrics provide complementary perspectives on overall and short-term autonomic variability, but their interpretation depends critically on recording duration and the specific physiological processes each metric emphasizes.

### Frequency-domain parameters

Frequency-domain analysis characterizes HRV by the speed at which fluctuations in heart rate occur. Rather than quantifying the overall amount of variability, these measures distinguish between rapid, beat-to-beat changes in heart rate and slower oscillatory patterns. In this way, frequency-domain metrics describe the temporal organization of autonomic modulation rather than its absolute magnitude. Although the full HRV spectrum includes multiple frequency ranges, this review focuses on HF power, LF power, and the LF:HF ratio, which remain the most commonly reported frequency-domain measures in cardiovascular research. While these metrics are often interpreted as reflecting different aspects of autonomic regulation, their physiological meaning depends strongly on measurement conditions and analytic assumptions. In specific, interpretation of lower-frequency components and ratio-based measures therefore requires particular caution, as their relationship to sympathetic and parasympathetic activity is complex and context dependent.

HF power reflects HRV occurring within the respiratory frequency range (approximately 0.15–0.40 Hz) and primarily indexes parasympathetic modulation of the sinoatrial node (4). Variability in this range corresponds to the normal acceleration and deceleration of heart rate that accompanies breathing, such that heart rate typically increases during inspiration and decreases during expiration under resting conditions (25). Because HF power is tightly coupled to this respiratory-linked modulation, it captures rapid, short-term fluctuations in heart rate over a few seconds and represents the frequency-domain analogue of vagally mediated variability (26). In general, higher HF power indicates stronger parasympathetic modulation, whereas lower HF power reflects reduced vagal influence and has been observed in settings of physiological stress and disease (27). Consistent with this physiology, HF power exhibits predictable diurnal variation, increasing during sleep and decreasing during wakefulness, reflecting normal circadian shifts in autonomic balance (28). Accordingly, HF power is most informative when interpreted alongside other parasympathetic indices and within a clearly defined measurement context.

LF power reflects HRV occurring in the range of approximately 0.04–0.15 Hz and represents slower oscillations in heart rate that unfold over several seconds (4). Unlike HF power, which is tightly linked to respiratory-driven parasympathetic modulation, the physiological determinants of LF power are more complex and heterogeneous. Historically, LF power was often interpreted as a marker of sympathetic activity, particularly when expressed in normalized units or as part of the LF: HF ratio. However, substantial experimental and clinical evidence has demonstrated that LF power does not reflect sympathetic modulation in isolation, as variability within the LF band is influenced by parasympathetic input even under resting conditions (29, 30). In autonomic blockade studies, LF power decreases with parasympathetic blockade and combined autonomic blockade, but not consistently with selective sympathetic blockade, challenging its use as a surrogate for cardiac sympathetic tone (31). Additional evidence indicates that LF power correlates strongly with baroreflex sensitivity and is largely independent of cardiac sympathetic innervation, with LF variability markedly reduced in states of impaired baroreflex function regardless of underlying sympathetic nerve integrity (32). This is further supported by findings from Cevese et al., who demonstrated that selectively reducing vasomotor tone, thereby impairing baroreflex input without directly affecting cardiac sympathetic outflow, resulted in a marked reduction or complete loss of LF power, even when arterial blood pressure was pharmacologically maintained under resting supine conditions (33). Together, these findings support interpreting LF power as a composite index influenced by multiple regulatory processes rather than a direct measure of sympathetic activity. While LF power may still provide insight into slower autonomic and reflex-mediated modulation of heart rate, its interpretation depends strongly on context and should not be used in isolation as evidence of sympathetic predominance.

The LF:HF ratio is a derived frequency-domain metric calculated as the ratio of LF to HF power and was historically proposed as an index of sympathovagal balance. Early interpretations assumed that LF power primarily reflected sympathetic activity, whereas HF power indexed parasympathetic modulation, leading to the inference that a higher LF:HF ratio indicated sympathetic predominance and a lower ratio indicated parasympathetic dominance (34). However, this interpretation relies on assumptions that are no longer physiologically supported. As discussed above, LF power does not represent sympathetic modulation in isolation and is substantially influenced by parasympathetic input and baroreflex-mediated mechanisms. Consequently, changes in the LF:HF ratio cannot be reliably interpreted as direct shifts in sympathovagal balance. Moreover, sympathetic and parasympathetic branches do not operate in strict reciprocity, and their simultaneous, independent modulation undermines the conceptual validity of ratio-based constructs that presume a linear trade-off between the two systems (35). Additional limitations arise from the mathematical properties of the LF:HF ratio itself. Because both LF and HF components are influenced by heart rate and measurement conditions, dividing these interdependent variables can amplify variability and distort interpretation (36). For example, the same LF:HF ratio value may arise from a doubling of parasympathetic activity, a halving of sympathetic tone, or concurrent changes in both, limiting the ability to draw meaningful inferences about the underlying autonomic physiology from the ratio alone (37). While the ratio remains widely reported due to historical precedent, its use as a marker of sympathovagal balance is not well supported, and caution is warranted when interpreting ratio-based constructs such as LF:HF values, particularly in isolation or without careful consideration of measurement context.

### Nonlinear and complexity-domain parameters

Time- and frequency-domain metrics summarize the magnitude of variability or its distribution across frequencies. Heart rate regulation, however, depends on the interaction of multiple physiological systems operating over different time scales, and a family of nonlinear methods—including Poincaré-plot geometry, detrended fluctuation analysis, entropy-based measures, and symbolic dynamics—has been developed to characterize the structure of R–R interval fluctuations rather than their amount (38). These methods differ considerably in how much information they add beyond the linear indices, and that variation is itself important for interpretation.

Poincaré plot analysis represents each R–R interval against the preceding interval, generating an ellipse whose short axis (SD1) and long axis (SD2) are taken to index short- and long-term variability, respectively. These descriptors are not, however, independent of conventional statistics. Brennan et al. showed that SD1^2^ equals half the squared standard deviation of successive differences (SDSD) and that SD2^2^ equals twice the squared SDNN minus half the squared SDSD, so that SD1 is a fixed multiple of SDSD — equal to RMSSD divided by the square root of two when the mean of the successive differences is zero — while SD2 is determined jointly by SDNN and SDSD rather than by SDNN alone. They concluded that the standard measures of Poincaré plot geometry quantify linear properties of the R–R interval sequence that existing HRV indices already specify, and appear insensitive to the nonlinear characteristics the plot was intended to display (39). The plot remains a useful visual representation of the interval series, but its ellipse-derived indices should not be reported as though they conveyed information independent of time-domain measures.

Detrended fluctuation analysis (DFA) quantifies the extent to which fluctuations at one time scale predict fluctuations at another, and is usually expressed as a short-term scaling exponent (α1) and a long-term exponent (α2). An exponent of approximately 0.5 denotes uncorrelated randomness resembling white noise, a value near 1.0 denotes long-range correlated, scale-invariant dynamics, and a value near 1.5 denotes random-walk behavior; healthy dynamics lie toward the correlated middle of this range, such that movement toward either extreme represents a loss of normal fractal organization (38). The prognostic relevance of the short-term exponent is well established. Among 446 survivors of acute myocardial infarction with an ejection fraction of 35% or less, a reduced α1 (below 0.75) carried a relative risk of death of 3.0 (95% confidence interval 2.5 to 4.2) and remained an independent predictor after adjustment for age, ejection fraction, New York Heart Association class, and medication; the authors concluded that fractal analysis of short-term R–R interval dynamics yielded more powerful prognostic information than traditional HRV measures (40).

Entropy-based measures quantify the irregularity or unpredictability of the R–R interval series. Approximate entropy and sample entropy assess regularity at a single time scale, whereas multiscale entropy extends this assessment across progressively coarse-grained scales; single-scale algorithms assign high entropy to uncorrelated random signals, whereas multiscale entropy separates such signals from the long-range correlated dynamics characteristic of health (41). Reduced complexity also carries prognostic information. In a pilot study of 40 patients with systolic heart failure followed for a mean of 684 days, a lower area under the multiscale entropy curve across longer time scales, derived from 24 h Holter recordings, identified those who subsequently died or underwent urgent heart transplantation and discriminated better than low-frequency power or DFA α1. In the same cohort, short-scale complexity and DFA α1 differed between patients receiving and not receiving beta-blockers whereas conventional linear indices did not, while the prognostic long-scale index was unrelated to beta-blocker use (42). These comparisons are cross-sectional and drawn from a small sample, but they illustrate that complexity indices may be sensitive to influences that linear measures do not register.

Nonlinear indices carry practical constraints that determine when they can be meaningfully interpreted. Several depend explicitly on recording length: detrended fluctuation analysis expects large time series, approximate entropy is heavily dependent on record length and returns values uniformly lower than expected for short records, and multiscale entropy loses reliability when applied to short time series (38). Most also require the analyst to make choices that materially affect the result, including the tolerance and embedding parameters for the entropy family and the position of the split between short- and long-term scaling for DFA (38). All are derived from normal-to-normal intervals and are therefore sensitive to the identification and editing of ectopic beats and artifacts, which influence both scaling and entropy estimates (38). For clinical purposes, nonlinear metrics are therefore best regarded as complementary indices, most informative when tracked within an individual over time or interpreted alongside linear measures, rather than as standalone diagnostic thresholds.

A boundary condition applies across these metric domains. Frequency-domain analysis in particular presupposes that the mechanisms modulating heart rate do not change during the recording, and standard recommendations accordingly specify that short-term recordings be obtained from a stationary system (34). This condition is not met during dynamic exercise, where the validity of several widely used metrics degrades substantially. Because HRV is increasingly recorded during exertion rather than only before and after it, we address this measurement context separately below.

## Measurement techniques and data acquisition context

Interpretation of HRV metrics depends not only on metric selection but also on how the underlying cardiac signal is acquired. HRV metrics are derived from sequences of R–R intervals, yet these intervals are not directly observed. They are estimated from physiological signals whose fidelity, resolution, and susceptibility to noise vary substantially across measurement techniques. As a result, the same HRV metric may reflect fundamentally different physiological information depending on the method used to capture the data. Measurement technique therefore plays a central role in how HRV findings should be interpreted. Factors such as sampling rate, signal quality, motion artifact, and recording duration determine how accurately R–R intervals are identified and how much of the underlying variability is preserved. These factors influence not only the reliability of HRV estimates, but also which components of autonomic variability can be meaningfully assessed. A method that is well suited for capturing short-term, beat-to-beat variability may be poorly suited for characterizing slower or more complex patterns of autonomic regulation.

Importantly, HRV metrics are not equally valid across all acquisition methods. The physiological interpretation of a given metric depends on whether the measurement system can reliably capture the temporal features that metric reflects. Failure to account for these constraints can lead to overinterpretation or inappropriate comparisons across studies using different devices or recording protocols. For this reason, HRV metrics should always be interpreted in the context of how the underlying data were acquired. With these considerations in mind, we review the most commonly used HRV acquisition methods and outline their respective strengths and interpretive limitations.

### 24 Hour electrocardiogram

Continuous 24 h electrocardiogram (ECG) is widely regarded as the reference standard for HRV assessment. Its technical strength lies in the direct measurement of cardiac electrical activity at high temporal resolution, which allows precise identification of R–R intervals, minimizes error from motion and signal noise, and enables reliable estimation of both time- and frequency-domain HRV metrics (4, 34). The principal advantage of 24 h ECG, however, lies in its ability to integrate HRV across multiple temporal scales, offering superior predictive value compared to short-term recordings (4, 43). By providing uninterrupted recordings across a full circadian cycle, 24 h ECG captures variability arising from daily activity, rest, and circadian organization, making it particularly well suited for global HRV indices such as SDNN, which derive much of their prognostic value from long-term variability (44). Consequently, long-term HRV assessment can detect subtle disruptions in physiological homeostasis that signal diminished adaptability and increased health risk. Patients with comparable clinical profiles often display markedly different HRV patterns during hospitalization, with lower HRV associated with blunted physiological reactivity and poorer overall health status (45, 46). These observations position 24 h HRV as a robust, non-specific marker of vulnerability, reflecting not only cardiovascular risk but also broader systemic dysfunction.

The integrative nature of 24 h HRV also introduces important interpretive considerations. Because extended recordings aggregate autonomic variability across diverse physiological and behavioral states, global HRV measures do not map to a single, discrete condition. Without explicit attention to measurement context, similar HRV values may reflect fundamentally different underlying states, limiting their interpretability. In addition, 24 h ECG monitoring is resource-intensive and less feasible for repeated or large-scale assessment, motivating the increasing use of shorter recordings and wearable devices that trade temporal completeness for scalability.

Despite these limitations, 24 h ECG provides the most comprehensive representation of autonomic variability and serves as the physiological anchor for HRV interpretation. Accordingly, HRV metrics derived from shorter or lower-fidelity recordings should not be treated as equivalent substitutes, but rather as context-specific windows into autonomic regulation shaped by the constraints of their measurement method.

### Short-term electrocardiogram

Short-term ECG recordings, typically recording over a matter of minutes, are commonly used for HRV assessment under standardized and controlled conditions. When signal quality is high, these recordings provide accurate R–R interval data and are well suited for quantifying HRV metrics that reflect rapid autonomic modulation, particularly parasympathetic indices such as RMSSD and HF power (47). Their feasibility and reproducibility make short-term ECG recordings practical for cross-sectional studies, experimental protocols, and repeated assessments in constrained clinical or laboratory environments.

By design, short-term recordings capture autonomic activity within a limited physiological window and do not reflect longer-term regulatory processes. As a result, they are not intended to characterize global autonomic adaptability or cumulative cardiovascular risk. Within this framework, short-term ECG-derived HRV should be interpreted as a measure of momentary autonomic state rather than an index of overall autonomic capacity.

### Chest strap heart rate monitors

Chest strap monitors estimate R–R intervals using electrodes positioned across the thorax, allowing detection of cardiac electrical activity without the need for full multi-lead ECG acquisition. Owing to their proximity to the heart and secure positioning, chest strap monitors minimize motion artifact and maintain signal stability during physical activity, making them well suited for ambulatory monitoring, exercise physiology studies, and longitudinal assessments conducted outside the clinical setting. When properly fitted and used under conditions of minimal motion artifact, high- quality models have demonstrated strong agreement with ECG-based measurements across both time- and frequency-domain HRV metrics (48). In comparative studies, chest straps have outperformed wrist-worn devices in accuracy, particularly during dynamic conditions; however, comfort and ease of use often lead users to favor wrist-based alternatives (49).

However, chest strap monitors remain sensitive to factors that influence electrode contact and signal stability, including body movement, respiration-related chest wall motion, perspiration, and strap positioning (50). Signal quality may degrade during high-intensity or irregular activity, limiting the reliability of derived HRV metrics under those conditions. In addition, chest strap recordings are typically short or intermittent and do not capture long-term variability related to circadian organization or cumulative physiological stress. Consequently, HRV metrics derived from chest straps should be interpreted as context-specific indicators of autonomic modulation rather than substitutes for extended ECG-based assessment.

Within this framework, chest strap monitors represent a pragmatic compromise between signal fidelity and feasibility. They provide a useful window into short-term autonomic dynamics in real-world environments, but their interpretive scope is defined by recording duration, activity context, and signal stability.

### Wrist-worn devices

Wrist-worn devices, including smartwatches and fitness trackers, are increasingly used for HRV assessment due to their convenience, comfort, and widespread adoption. These devices typically estimate inter-beat intervals using photoplethysmography (PPG), which infers cardiac timing from pulse wave-induced blood volume changes in the peripheral microvasculature. Their unobtrusive design enables passive HRV monitoring during daily activities and sleep, facilitating large-scale data collection in free-living environments.

While generally yielding acceptable accuracy for time-domain HRV metrics under typical resting conditions, wrist-worn devices can suffer from reduced reliability during high-intensity activities, where motion artifacts may distort readings (51–53). In a comparative evaluation of five wrist-worn devices during aerobic exercise, measurement accuracy declined with increasing movement, leading the authors to recommend electrode-based chest monitors when precision is required (54). Inter-device variability further complicates interpretation, as proprietary signal processing and metric derivation algorithms differ across manufacturers, limiting transparency and comparability between platforms. Recent advances, including multi-wavelength PPG sensors and machine learning–based artifact correction, aim to improve signal fidelity across a broader range of conditions, though validation remains ongoing (55).

In practice, wrist-worn devices are best suited for tracking relative changes in HRV within individuals over time rather than for precise physiological inference or cross-device comparison. Their primary strength lies in scalability and longitudinal monitoring under resting or sleep conditions, not in detailed characterization of autonomic regulation. Accordingly, wearable-derived HRV metrics should be interpreted with explicit attention to recording context, device-specific processing, and alignment between the reported metric and the physiological process it is intended to reflect.

Table 1 summarizes HRV metrics and measurement modalities.

**Table 1:** Heart rate variability metrics and measurement modalities: physiological significance, optimal acquisition, and interpretive considerations.

Item	What it reflects	Best acquisition/practical notes	Interpretation pearls	Key limitations
24 h ECG (reference standard)	Captures the full spectrum of autonomic fluctuations, including circadian rhythms and responses to daily activities; provides greater predictive power than short-term assessments.	Preferred for risk stratification and long-term HRV; provides required resolution for R-R intervals used to derive HRV metrics.	Long-term HRV can reveal risk-related homeostatic disruptions and reduced adaptability (blunted reactivity) in poorer health states.	Short-term recordings may miss daily/circadian variation; ultra-low-frequency band typically requires extended monitoring for accuracy.
Chest strap monitors (electrode-based)	Practical alternative to 24 h ECG, capturing R-R intervals similar to single-lead ECG; allows monitoring across daily activities/exercise.	High-quality models (e.g., Polar H10) show strong correlation with ECG across metrics; less motion artifact due to proximity to the heart.	When precision is needed, chest straps may be preferable to wrist devices (greater accuracy in comparative studies).	Comfort/wearability may drive preference toward wrist devices despite accuracy advantages.
Wrist-worn devices (PPG-based)	Uses photoplethysmography (PPG) to estimate heart rate and derive HRV; enables continuous monitoring during daily activity and sleep.	Generally acceptable accuracy in typical conditions; technology improvements (multi-wavelength PPG + machine learning) aim to reduce inaccuracies.	Useful for tracking HRV trends in everyday, non-exercise settings; democratizes access.	Reliability can drop during high-intensity activity due to motion artifacts; manufacturer algorithm differences can cause inter-device inconsistency.
SDNN (time-domain)	A comprehensive index of overall HRV (combined sympathetic + parasympathetic influences over extended period); circadian variation with higher nocturnal parasympathetic activity.	Can be calculated from short-term recordings, but 24 h SDNN captures short- and long-term fluctuations (respiration, movement, emotion, activity/rest transitions).	24 h SDNN has been described as the gold standard for cardiac risk stratification with prognostic value for cardiovascular morbidity/mortality.	Does not differentiate sympathetic vs. parasympathetic contributions → interpret with other parameters.
RMSSD (time-domain)	Marker of vagal activity; reflects high-frequency, beat-to-beat parasympathetic-driven variability.	Reliably measured in 5 min recordings; shorter recordings of 1–2 min, and in some contexts 30 s, may correlate reasonably with 5 min values under resting conditions	Higher RMSSD generally indicates strong vagal tone and rapid adaptability; complementary to SDNN (total HRV vs. parasympathetic influence).	Short-term metric; best interpreted with context and alongside longer-term measures (e.g., SDNN).
pNN50 (time-domain)	Percentage of adjacent N-N intervals differing by >50 ms; primarily influenced by high-frequency components driven by parasympathetic influences.	Practical for monitoring short-term autonomic fluctuations (dynamic vagal tone changes).	Highlights quick adaptability of autonomic nervous system in settings where dynamic changes matter.	Same general constraints as short-term vagal metrics; interpret within measurement context.
HF Power (frequency-domain)	Captures rapid fluctuations tied to breathing (respiratory sinus arrhythmia); 0.15–0.4 Hz; primarily influenced by parasympathetic activity.	HF metrics can be reliably captured in recordings as short as 1–5 min.	Higher HF generally indicates a well-functioning parasympathetic nervous system and recovery capacity; increases at night with circadian shift to rest.	Strongly influenced by breathing-related physiology; interpret with context of measurement conditions.
LF Power (frequency-domain)	Slower fluctuations 0.04–0.15 Hz; reflects mixed sympathetic + parasympathetic influences; linked to baroreceptor activity and longer rhythms.	LF components typically require a longer recording (compared with HF components) for accurate assessment.	Higher LF may reflect preserved autonomic regulatory capacity; lower LF suggests reduced autonomic responsiveness (interpretation varies and is context-dependent).	Interpretation can vary; avoid oversimplifying LF as purely sympathetic.
Poincaré indices—SD1/SD2 (nonlinear)	Geometric display of each R–R interval against the preceding one; SD1 is taken to index short-term and SD2 long-term variability.	Any recording adequate for RMSSD/SDNN; value lies largely in visual inspection of plot shape.	Useful as a visual display of the interval series; the ellipse indices restate linear time-domain information.	SD1 equals RMSSD divided by the square root of two, and SD2 is determined jointly by SDNN and SDSD — not independent of time-domain measures.
DFA α1/α2 (nonlinear)	Fractal scaling of R–R fluctuations across time scales; short-term (α1) and long-term (α2) correlation exponents.	Expects long time series; the analyst must choose where the short- and long-term scaling regions are separated.	α = 0.5 denotes uncorrelated randomness, = 1.0 long-range correlation, = 1.5 random walk. Reduced α1 (<0.75) predicted mortality after myocardial infarction with reduced ejection fraction.	Requires normal-to-normal intervals and is sensitive to editing of ectopic beats; unsuited to short recordings.
Entropy measures—ApEn/SampEn/MSE (nonlinear)	Irregularity and unpredictability of the R–R series; multiscale entropy extends assessment across coarse-grained time scales.	Long recordings preferred (24 h Holter for multiscale entropy); parameters (m, r, scale range) must be reported.	Reduced long-scale complexity has been associated with death or urgent transplantation in heart failure.	Values depend on the tolerance and embedding parameters chosen; reliability falls in short recordings.

HRV, heart rate variability; ECG, electrocardiogram; SDNN, standard deviation of All N-N intervals; RMSSD, root mean square of successive differences; pNN50, percentage of adjacent N-N intervals differing by more than 50 milliseconds; HF, high-frequency; LF, low-frequency. SD1, standard deviation perpendicular to the line of identity; SD2, standard deviation along the line of identity; DFA, detrended fluctuation analysis; α1, short-term scaling exponent; α2, long-term scaling exponent; ApEn, approximate entropy; SampEn, sample entropy; MSE, multiscale entropy.

## Temporal context of heart rate variability assessment

### Assessing autonomic stability: single-timepoint vs. longitudinal HRV measurement

HRV is frequently reported as a single-timepoint measure, yet its physiological meaning is inherently dynamic. Because HRV is highly sensitive to transient influences such as sleep, stress, illness, and recent activity, isolated measurements primarily reflect momentary autonomic state rather than stable autonomic function. As a result, single-timepoint HRV values may over- or underestimate baseline autonomic capacity depending on the context in which they are obtained.

In contrast, repeated measurements provide a fundamentally different perspective by characterizing autonomic stability over time. Longitudinal HRV patterns allow differentiation between transient perturbations and persistent dysregulation, revealing trajectories of adaptation, recovery, or decline that are not apparent in cross-sectional data. Two individuals with similar HRV values at a single assessment may exhibit markedly different longitudinal patterns, with distinct implications for resilience and risk. Accordingly, HRV should not be interpreted as a stable trait unless its reproducibility has been demonstrated. Single-timepoint measurements may be appropriate for assessing acute physiological state, but longitudinal assessment is better suited for evaluating autonomic stability, recovery, and longer-term risk.

### Circadian timing: interpreting HRV across the Sleep–Wake cycle

HRV is often assessed during nocturnal sleep, as this period provides a relatively stable physiological environment with reduced behavioral and environmental confounding. Under these conditions, parasympathetic influence is more prominent, allowing clearer assessment of vagally mediated HRV indices (56). However, sleep is not a uniform physiological state. Autonomic balance shifts across the sleep–wake cycle, with parasympathetic predominance and higher HRV during non–rapid eye movement sleep and vagal withdrawal with greater sympathetic activation during rapid eye movement sleep (57, 58). Accordingly, a single nighttime HRV value reflects a composite of physiological influences rather than a discrete measure of autonomic function, and must be interpreted in the context of sleep stage distribution and sleep quality.

The assumption of stable, restorative sleep underlying many HRV studies is frequently violated, with important implications for HRV interpretation. In a study comparing individuals with insomnia to healthy controls during sleep onset, insomnia was associated with higher heart rates and reduced vagally mediated HRV indices, reflecting pre-sleep hyperarousal rather than intrinsic autonomic dysfunction (59). Similarly, experimental sleep restriction has been shown to increase heart rate and reduce HRV indices, including SDNN and HF power, consistent with a shift toward sympathetic dominance during the night (60). Moreover, a longitudinal study of repetitive sleep restriction demonstrated that HRV recovers gradually over multiple nights following restoration of normal sleep, highlighting that autonomic responses to sleep disruption evolve over time rather than resolving immediately (61). These observations indicate that acute deviations in nighttime HRV may reflect transient sleep-related physiological states rather than fixed autonomic impairment. When persistent, however, such disturbances may contribute to sustained autonomic dysregulation, such that meaningful interpretation of HRV requires consideration of patterns over time rather than reliance on single-night measurements.

Consistent with this, large population studies have linked abnormal sleep duration and disrupted sleep with increased cardiovascular morbidity and mortality (62, 63). In this context, the influence of sleep disturbances on HRV should not be viewed solely as a source of interpretive complexity, but also as clinically meaningful information when assessed longitudinally. Single-night measurements may obscure these patterns, therefore repeated HRV assessments are better suited to capturing the sustained physiological effects of chronic sleep patterns.

## Behavioral and environmental context of heart rate variability

HRV is highly sensitive to behavioral and environmental influences, which shape autonomic regulation on a moment-to-moment basis. Factors including physical activity, diet, psychological stress, substance use, and environmental exposures can induce rapid and substantial changes in HRV independent of underlying cardiovascular health. As a result, HRV measurements obtained in uncontrolled or real-world settings reflect not only intrinsic autonomic function, but also the immediate behavioral and environmental context in which they are recorded. Many of these factors exert both acute and sustained effects on autonomic regulation, complicating efforts to distinguish transient, state-dependent variation from stable, trait-level differences in HRV. Without explicit attention to context, differences in HRV may be misinterpreted as reflecting physiological impairment rather than normal adaptive responses to external demands. Accordingly, interpretation of HRV requires careful consideration of the behavioral and environmental conditions surrounding measurement, as well as the habitual patterns of behavior that shape baseline autonomic function over longer time scales. The following sections review the principal behavioral and environmental influences relevant to HRV interpretation, with attention to both acute measurement effects and longer-term modulators of autonomic regulation.

### Physical activity and cardiorespiratory fitness

Physical activity is among the most influential behavioral determinants of HRV, with both acute and sustained effects on autonomic regulation. Acutely, exercise produces a transient withdrawal of parasympathetic tone and an increase in sympathetic activity during the active period, followed by a gradual restoration of vagal modulation during recovery. The rate of post-exercise parasympathetic reactivation is itself shaped by exercise intensity, duration, and individual cardiorespiratory fitness, with delayed recovery observed after high-intensity efforts (35). As a result, HRV measurements obtained in the hours following exercise may reflect ongoing autonomic recovery rather than baseline function and should be interpreted with caution in this window. Beyond these immediate effects, regular physical activity and higher cardiorespiratory fitness are consistently associated with elevated HRV across multiple indices. A 2024 meta-analysis of 16 randomized controlled trials in healthy adults demonstrated that structured exercise training significantly increased SDNN, RMSSD, and HF power compared with non-training controls, with effect sizes that were largest for vagally mediated indices (64). A subsequent network meta-analysis suggested that aerobic training and combined training modalities may produce the most consistent improvements in vagally mediated HRV, although substantial heterogeneity across studies limits firm conclusions about the optimal modality (65). Observational data further support a dose-response relationship between higher-intensity physical activity and parasympathetic HRV indices, with one large cross-sectional study identifying measurable benefit up to approximately 21 min per week of very vigorous activity, beyond which no additional autonomic gain was observed (66).

The association between physical activity and HRV is, however, highly context dependent. In contrast to leisure-time exercise, occupational physical activity performed at high intensity over prolonged periods has been associated with lower rather than higher nocturnal HRV, suggesting that not all forms of physical exertion confer equivalent autonomic benefit (67). Conversely, sedentary behavior has been independently linked to reduced HRV across diverse populations, including reductions in SDNN, RMSSD, and HF power (68). These observations indicate that the autonomic profile measured at a given moment reflects not only acute activity status but also cumulative habitual exposure to physical activity or inactivity.

For interpretation, physical activity introduces multiple, partially independent sources of variation in HRV. Recent exercise may transiently depress HRV through ongoing parasympathetic recovery, while chronic differences in fitness, occupational physical demands, and sedentary behavior may produce sustained baseline differences between individuals. HRV recordings intended to characterize resting autonomic state should therefore be obtained after a standardized period of rest, and inferences regarding autonomic health should consider both acute activity status and habitual physical activity patterns surrounding the measurement.

### HRV measured during exercise

The preceding subsection concerns HRV measured before and after exercise. HRV recorded during exercise itself poses a distinct interpretive problem, encountered with increasing frequency as chest strap monitors and wearable devices are used in cardiac rehabilitation and training settings. Three features of the exercising state limit the validity of conventional metrics. First, the R–R interval series during exercise is non-stationary: heart rate drifts even at constant external workload, so that analyses depend on detrending or on time-varying spectral methods rather than on the stationarity assumed by conventional spectral estimation (34, 35). Second, respiratory frequency rises with intensity and may exceed the conventional HF band (0.15–0.40 Hz), so that this band no longer captures respiratory-linked variability; investigators have consequently adopted a wide range of alternative upper limits, and the physiological interpretation of HF power during exercise becomes correspondingly uncertain (35). Third, vagal withdrawal causes RMSSD to fall more rapidly than SDNN with rising intensity, reaching a minimum below 5 ms at approximately 50 to 60% of maximal oxygen uptake, beyond which it changes little with further increases in workload (35). The time- and frequency-domain metrics reviewed earlier should therefore not be interpreted during dynamic exercise as they are at rest; low values in this setting reflect the expected physiology of exertion rather than autonomic impairment.

Nonlinear indices remain interpretable in this setting, as they depend neither on spectral decomposition nor on the absolute magnitude of variability. The short-term scaling exponent DFA α1 declines progressively with rising workload as beat-to-beat dynamics shift from correlated behavior toward uncorrelated randomness. In a study of 20 patients with chronic heart failure, the intensity at which α1 fell to 0.5 showed good agreement with the second ventilatory threshold for oxygen uptake, heart rate, and power output, with intraclass correlation coefficients between 0.81 and 0.86, whereas the intensity at which α1 fell to 0.75 corresponded only modestly with the first ventilatory threshold and showed wide limits of agreement (69). This implies that a nonlinear index continues to vary systematically with workload at intensities at which RMSSD and HF power have already reached their floor.

Beyond threshold detection, HRV measured during exercise may carry prognostic information that resting measurement does not. In 77 patients with advanced heart failure with reduced ejection fraction, entropy-based HRV was assessed at rest, during early exercise, and at peak exercise. Conventional time-domain indices and absolute spectral powers showed the expected workload-dependent attenuation and did not differ by outcome, whereas information-theoretic measures obtained during early, submaximal exercise distinguished the 17 patients who subsequently reached a composite endpoint of cardiac death, heart transplantation, or left ventricular assist device implantation from those who did not, with lower conditional entropy and higher self-entropy in the former group; early-exercise self-entropy remained independently associated with this endpoint after adjustment for clinical and exercise-derived variables, although the confidence interval was wide given the small number of events (70). No comparable separation was evident at rest or at peak exertion. Read alongside the resting-state association between reduced complexity and outcome in heart failure (42), this suggests that submaximal exertion may function as a physiological stress test that unmasks autonomic impairment not apparent under resting conditions, although the finding requires replication in larger and less selected cohorts.

For interpretation, the exercising state should be treated as a distinct measurement context rather than a continuation of the resting one. Values obtained during exercise are not comparable to resting values, and workload, relative intensity, and metric domain must all be specified for such a recording to be interpretable. Where the clinical question concerns autonomic capacity rather than momentary autonomic state, resting or post-exercise recovery measurement remains more appropriate; where HRV is assessed during exercise, nonlinear indices are at present better suited than linear ones, though the supporting evidence base remains limited and largely confined to research settings.

### Dietary patterns and postprandial state

Dietary intake influences HRV through both acute postprandial mechanisms and longer-term effects on metabolic and inflammatory status. Acutely, food ingestion produces measurable changes in autonomic regulation during the digestive period, with shifts in HRV that vary by meal composition and size. In a recent controlled experiment, high-carbohydrate and high-fat diets produced distinct postprandial autonomic profiles, with reductions in vagally mediated HRV indices accompanying glycemic excursions following meals (71). These transient effects suggest that HRV recordings obtained within a few hours of a substantial meal may not reflect resting autonomic function, and that standardization of fasting status is important for between-subject comparison.

Over longer time scales, dietary patterns have been associated with sustained differences in HRV. Adherence to a Mediterranean dietary pattern has been linked to higher HRV in both time- and frequency-domain indices, with one twin study reporting substantially greater HRV in men with the highest dietary adherence relative to those with the lowest (72). Higher overall diet quality, characterized by greater intake of fruits, vegetables, and whole grains, has similarly been associated with more favorable HRV profiles in observational cohorts, although residual confounding by lifestyle and metabolic factors limits causal inference (73). Conversely, dietary patterns high in saturated fat and refined carbohydrates have been associated with lower HRV, plausibly mediated by their downstream effects on systemic inflammation, insulin resistance, and adiposity (74).

A related interpretive consideration is adiposity itself, which often reflects the cumulative consequence of dietary and activity patterns over time. Excess body fat, particularly when distributed centrally, has been associated with lower HRV across multiple time- and frequency-domain indices, with central adiposity measures such as waist circumference showing stronger associations with HRV than overall body mass index (75). These findings suggest that HRV reflects not only acute dietary effects but also longer-term metabolic and structural consequences of habitual dietary patterns. HRV recordings are sensitive to both the immediate prandial state and the broader nutritional context in which they are obtained. Standardization of fasting status and time since last meal can reduce variability attributable to acute dietary effects, while attention to habitual dietary patterns and body composition may help distinguish reversible nutritional influences from more stable autonomic differences between individuals.

### Psychological stress and affective states

Psychological state exerts a powerful influence on HRV, both acutely and chronically. Acute psychological stress consistently produces sympathetic activation and parasympathetic withdrawal, manifesting as reduced RMSSD, HF power, and other vagally mediated indices (76). These changes can be substantial in magnitude and rapid in onset, such that even brief stressors imposed in the minutes preceding HRV measurement may shift values away from a true resting baseline. A meta-analysis of 37 studies confirmed that HRV reliably reflects both acute and chronic psychological stress, supporting its sensitivity as a physiological marker of autonomic strain (77).

Beyond acute responses, sustained psychological distress is associated with persistent reductions in HRV. Both anxiety disorders and major depressive disorder have been associated with reduced HRV in large pooled analyses, with diminished RMSSD and HF power consistently reported across diverse patient populations (78, 79). A recent population-based study of nearly 10,000 adults further identified an association between low daytime RMSSD and greater depressive symptomatology, suggesting that HRV may capture autonomic correlates of subclinical mood disturbance that are not always evident through clinical assessment alone (80).

The bidirectional relationship between psychological state and HRV introduces meaningful interpretive complexity. Reduced HRV in an individual under acute or chronic stress may reflect a normal adaptive response rather than fixed cardiovascular dysfunction, particularly when measurements coincide with emotionally salient periods. Conversely, individuals with persistent psychiatric conditions may exhibit lower baseline HRV that is not attributable to primary cardiovascular pathology, but to sustained autonomic effects of their underlying psychological state. Without explicit consideration of these factors, HRV may overestimate cardiovascular risk in psychologically vulnerable populations or underestimate it in individuals whose values are transiently elevated during low-stress measurement periods. Accordingly, accurate interpretation of HRV requires consideration of the individual's psychological state at the time of measurement and, where relevant, recent exposure to stressors. In research contexts, this may warrant concurrent assessment of state anxiety, depressive symptoms, or perceived stress alongside HRV recording. In clinical or wearable-based monitoring, attention to longitudinal patterns may help distinguish transient, stress-related fluctuations from sustained autonomic changes of clinical significance.

### Substance use

Commonly used substances, including alcohol, caffeine, and tobacco, exert measurable effects on HRV that may persist for hours to days following consumption. Because these substances are widely used and often unrecorded in HRV measurement protocols, their effects represent a substantial and frequently underappreciated source of interpretive variability.

Alcohol intake disrupts autonomic balance in a dose-dependent manner, with higher consumption producing greater reductions in HF power and other parasympathetic HRV indices. These effects typically emerge within approximately 30 to 60 min of ingestion, parallel rising blood alcohol levels, and persist for several hours (81). The autonomic effects of alcohol extend well beyond the period of acute intoxication. In a large real-world observational study, evening alcohol consumption was associated with reductions in vagally mediated HRV indices and elevated heart rate during subsequent sleep, indicating that autonomic effects persist into the nocturnal recovery period (82). Among individuals with alcohol use disorder, resting HRV is persistently reduced and recovers gradually over the course of sustained abstinence, although normalization may require many months (83). For interpretation, HRV measurements obtained within 24 h of substantial alcohol intake, or in individuals with active or recent heavy alcohol use, should be interpreted with awareness that both acute and chronic alcohol-related effects may meaningfully depress observed values.

Caffeine, the most widely consumed psychoactive substance globally, also influences HRV, although the magnitude and direction of its effects have varied across studies. Acutely, caffeine blocks adenosine receptors and increases sympathetic outflow, yet the resulting HRV response is highly context dependent (84). In nonhabitual users, acute caffeine ingestion has been associated with reductions in vagally mediated HRV indices, whereas habitual consumers may show no detectable change, consistent with tolerance and baroreflex-mediated compensation (85, 86). Other studies have reported transient increases in HF power following caffeine ingestion, with effects modulated by dose, posture, and underlying cardiovascular health (87–89). In the large ELSA-Brasil cohort, an initial inverse association between heavy coffee consumption and vagally mediated HRV was attenuated to non-significance after adjustment for lifestyle and clinical confounders (90). Given this heterogeneity, recent caffeine consumption should be documented prior to HRV measurement, as it is when interpreting heart rate or blood pressure, and findings should not be over-interpreted in individuals whose caffeine status is unknown.

Tobacco use produces some of the most consistent and pronounced effects on HRV among modifiable substances. Acute cigarette smoking results in immediate decreases in vagally mediated HRV indices, including HF power, RMSSD, and pNN50, within minutes of inhalation, reflecting rapid sympathetic activation and parasympathetic withdrawal (91, 92). These effects extend beyond the period of acute exposure. In a population-based analysis of nearly 5,000 adults from the CHRIS cohort, current smokers demonstrated dose-response reductions in SDNN, RMSSD, total power, LF power, and HF power per 10 grams of tobacco smoked per day, independent of cardiometabolic conditions and HRV-modifying medications (93). In the same study, past smokers exhibited HRV levels comparable to or higher than never smokers, suggesting that the autonomic effects of smoking may be at least partially reversible following cessation, although the time course and completeness of recovery likely depend on cumulative exposure and concurrent lifestyle factors (93). Electronic cigarette use has also been associated with reductions in vagally mediated HRV in systematic reviews, although typically of smaller magnitude than conventional cigarette smoking (94). Even passive exposure may meaningfully affect HRV. In the SAPALDIA cohort, lifelong non-smokers exposed to environmental tobacco smoke for more than two hours daily showed lower total power, LF power, and ultra-low-frequency (ULF) power, alongside higher resting heart rate, compared with unexposed peers (95).

In careful interpretation, it must be recognized that HRV measurements obtained in individuals with recent or chronic substance exposure may reflect substance-related autonomic effects superimposed on intrinsic cardiovascular regulation. Without explicit consideration of substance history, depressed HRV in such individuals may be misattributed to primary cardiovascular dysfunction. Documentation of recent substance use, including time since last consumption and habitual patterns, is therefore essential for accurate HRV interpretation.

### Environmental exposures and ambient conditions

Environmental factors can meaningfully influence HRV independent of intrinsic cardiovascular function, with effects that may be acute, cumulative, or both. Among these, ambient air pollution has emerged as a particularly well-characterized exposure. In a meta-analysis of 33 panel studies, short-term increases in fine particulate matter (PM2.5) were associated with measurable reductions in HRV, with consistent decreases observed across SDNN, RMSSD, HF power, and LF power (96). An earlier meta-analysis encompassing more than 18,000 individuals similarly reported inverse associations between particulate exposure and multiple HRV indices, supporting autonomic dysregulation as a plausible mechanism linking air pollution to cardiovascular disease (97). More recent work has extended these findings to ultrafine particles, with a systematic review reporting reductions in vagally mediated HRV within hours of exposure, although effect estimates were heterogeneous across studies (98). These observations indicate that HRV measurements obtained in regions or settings with elevated ambient pollution may be influenced by exposure-related autonomic effects that are difficult to disentangle from intrinsic cardiovascular regulation.

Ambient temperature represents a second environmental influence on HRV. Both heat and cold exposure produce measurable changes in autonomic regulation, although the magnitude and direction of these effects depend on exposure duration, intensity, and individual characteristics. In an experimental study of short-term whole-body cold exposure under conditions comparable to subarctic winter, both LF and HF power increased while heart rate decreased, consistent with facial cooling–induced vagal activation (99). By contrast, more extreme or prolonged cold and heat exposures have been associated with shifts toward sympathetic predominance, suggesting that the autonomic response to thermal stress is non-linear and condition specific. These observations imply that HRV measurements obtained in non-thermoneutral environments may not be directly comparable to those obtained under controlled laboratory conditions.

Acute physiological state at the time of measurement, including hydration status, may further modify HRV. In experimental work, dehydration following exercise in the heat reduced overall HRV and blunted post-exercise oscillations in LF and HF power, indicating that fluid status independently influences autonomic stability and short-term variability (100). Although such effects are most pronounced in the context of exercise and heat stress, they reinforce the broader principle that transient physiological perturbations can shift HRV away from true resting values.

Environmental exposures introduce both immediate and cumulative sources of variation in HRV and must be thus carefully considered when interpreting HRV data. Acute changes in ambient air quality, temperature, or hydration status at the time of measurement may shift HRV away from intrinsic values, while sustained exposure to high-pollution environments may contribute to chronic autonomic alterations that are not reflective of modifiable individual behavior. Awareness of the environmental context surrounding measurement is therefore essential when interpreting HRV in real-world, occupational, or longitudinal settings, particularly as wearable-based monitoring increasingly captures HRV across diverse and uncontrolled environments.

## Influence of host factors, medications, and comorbidities on HRV interpretation

HRV metrics are not only influenced by intrinsic autonomic function, but also by non-modifiable host characteristics such as age and sex, by medications, and by underlying comorbidities. Drugs that affect heart rate, cardiac contractility, vascular tone, or autonomic balance (such as beta-blockers, renin–angiotensin system inhibitors, and muscarinic receptor blockers) may alter HRV parameters or modify their clinical meaning (34, 101). Similarly, diseases such as heart failure, ischemic heart disease, diabetes, hypertension, obesity, and other systemic illnesses can independently change HRV through autonomic dysfunction, inflammation, altered hemodynamics, or end-organ disease (34, 102–104). Therefore, HRV should not be interpreted in isolation; medication exposure and comorbidity burden should be considered when comparing patients, defining abnormal values, or using HRV for prognostic purposes.

### Age and sex

Age and sex are the most consistent non-modifiable determinants of HRV and should be accounted for before any value is judged abnormal. HRV declines with age independently of disease, but the decline is measure dependent. Among 260 healthy subjects aged 10 to 99 years studied with 24 h recordings, SDNN and SDANN declined only gradually, reaching 60% of second-decade values by the tenth decade, whereas the vagally mediated indices pNN50 and RMSSD fell far more steeply, to 24% and 47% of baseline respectively by the sixth decade, and then stabilized (105). A comparable pattern was observed in the family-based Baependi Heart Study, in which SDNN and SDANN decreased linearly with age while RMSSD and pNN50 followed a U-shaped distribution, with a nadir at approximately 53 years and a reversal above the age of 60 (106). Age-referenced interpretation is therefore necessary, and a single normal range applied across adulthood will misclassify both younger and older individuals.

Sex differences are equally well documented, but they are metric specific rather than uniform, and are frequently summarized more simply than the evidence allows. In a meta-analysis of 172 studies comprising 63,612 healthy adults, women showed higher high-frequency power, in both absolute and normalized units, together with a higher mean heart rate; the same analysis found lower total spectral power, lower low-frequency power, a lower LF: HF ratio, and lower SDNN in women, and no consistent sex difference in RMSSD or pNN50 (107). A female advantage is therefore specific to the respiratory-frequency component rather than a general feature of vagally mediated HRV. Twenty-four-hour recordings reinforce this qualification. Among the 260 healthy subjects spanning nine decades, HRV was lower in women than in men for all time-domain measures below the age of 30, with the difference narrowing thereafter and disappearing beyond the age of 50 (105); and among 543 healthy participants in the Baependi Heart Study, men showed higher SDNN, SDANN, and pNN50, with higher vagal indices in women confined to the 40 to 49 and 50 to 59 year age groups (106). Age and sex therefore interact, and the direction of an apparent sex difference depends on which metric is examined.

For interpretation, age- and sex-referenced comparison should precede any inference about autonomic health, and normative values should be drawn from studies matched to the recording duration, metric, and population in question. Because the direction of the sex difference is not uniform across metrics, a normative comparison drawn from a mismatched index may distort a conclusion rather than merely widen its uncertainty.

### Beta-blockers

Beta-blockers can significantly influence HRV and should therefore be considered when interpreting HRV metrics. Beta-blockers may increase HRV by reducing sympathetic drive and improving cardiac autonomic balance, particularly in coronary artery disease or heart failure populations, and this may contribute to the protective effects of β-blockade (108, 109). In patients with coronary artery disease, Niemelä et al. showed that both atenolol and metoprolol significantly increased HRV, including HF power and RMSSD, suggesting that improved heart rate variability associated with beta-blocker therapy may represent one of the mechanisms underlying their beneficial effects in ischemic heart disease (108). In decompensated heart failure, Aronson and Burger found that HRV indices were markedly suppressed overall, but were substantially higher among patients receiving beta-blockers (110). Specifically, beta-blocker therapy was associated with higher pNN50, RMSSD, total power, ULF power, and HF power, supporting improved autonomic regulation during states of high sympathetic stress (110). In chronic heart failure with reduced ejection fraction, Bullinga et al. reported that carvedilol significantly increased SDNN, RMSSD, pNN50, total frequency power, very-low-frequency (VLF) power, and HF power (109). Importantly, changes in HRV correlated positively with improvement in left ventricular ejection fraction and negatively with coronary sinus norepinephrine levels, suggesting that HRV improvement may reflect both hemodynamic recovery and reduced sympathetic activation (109). Therefore, beta-blocker exposure should be accounted for when comparing HRV between patients or interpreting HRV as a marker of autonomic dysfunction or prognosis.

### Anticholinergic agents

Medications with anticholinergic properties can also influence HRV. Muscarinic receptor blockers, such as atropine, scopolamine, or related anticholinergic agents, can reduce vagally mediated HRV components by inhibiting parasympathetic input to the sinoatrial node (111). This is clinically important because reduced HRV may reflect pharmacologic vagal blockade rather than intrinsic autonomic dysfunction. Low dose anti-muscarinic paradoxically enhance parasympathetic (vagal) tone and increase the HRV, potentially through centrally mediated mechanisms (34, 111). A study by Huang et al. conducted on patients with schizophrenia demonstrated that those receiving antipsychotics with high muscarinic affinity (such as chlorpromazine and olanzapine) had significantly reduced HRV parameters (LF, HF, and total power) compared with those receiving low muscarinic affinity agents (such as haloperidol and risperidone), who exhibited relatively higher HRV and better preserved autonomic modulation (112).

### Renin–angiotensin system inhibitors

Renin–angiotensin system inhibitors may also modify HRV and should be considered when interpreting autonomic metrics. Angiotensin-converting enzyme (ACE) inhibitors such as lisinopril or quinapril, and Angiotensin II receptor blockers (ARBs) such as losartan or valsartan, have been associated in several studies with favorable changes in autonomic modulation, and this probably contribute to the cardioprotective effects of these medications in heart failure and ischemic heart disease (101, 113, 114). In a randomized study by De Tommasi et al. in patients with chronic heart failure, both lisinopril and valsartan produced comparable effects on cardiac vagal control of heart rate, while valsartan produced a greater reduction in plasma norepinephrine levels, suggesting stronger modulation of sympathetic activity (113). A study by Özdemir et al. in patients with ischemic cardiomyopathy and NYHA class II–III systolic heart failure showed that adding losartan to background beta-blocker and ACE-inhibitor therapy for 12 weeks significantly improved most HRV parameters, including SDNN, SDANN (standard deviation of the average NN intervals), triangular index, RMSSD, NN50, and SDNN index, and also improved heart rate turbulence indices (101). In post-myocardial infarction patients, Kontopoulos et al. reported that quinapril, lisinopril, and captopril improved frequency-domain HRV indices related to vagal tone, whereas enalapril and cilazapril did not show significant effects (114). Similarly, in patients with diabetic autonomic neuropathy, chronic quinapril therapy increased total HRV and parasympathetic-related indices, including pNN50, RMSSD, and HF power (115). These findings suggest that ACE inhibitors and ARBs may favorably influence sympathovagal balance, potentially contributing to their cardioprotective effects in heart failure, ischemic heart disease, and diabetes-related autonomic dysfunction.

Therefore, HRV values should not be interpreted as purely reflecting intrinsic autonomic function; medication exposure, drug class, dose, and clinical indication should be accounted for when comparing patients or using HRV prognostically. Moreover, chronic diseases can substantially alter HRV and therefore must be considered when interpreting HRV metrics.

### Diabetes mellitus

Diabetes mellitus, particularly when complicated by autonomic neuropathy, is associated with reduced HRV due to impaired parasympathetic and sympathetic regulation. In this population, HRV assessment has long been combined with cardiovascular autonomic reflex testing—metronomic breathing, Valsalva, and standing maneuvers—alongside short- and long-term time- and frequency-domain analysis; reference values for the reflex-test ratios have been derived from healthy adults across several centers, with reported coefficients of variation between approximately 4% and 7% on repeat testing (12). The interpretive considerations discussed here are intended to complement rather than replace that methodological framework. A study by Tarvainen et al. demonstrated that patients with type 2 diabetes had significantly lower HRV parameters compared with healthy controls, supporting the presence of cardiac autonomic dysfunction even before overt clinical autonomic neuropathy (116). The same study showed that higher blood glucose and HbA1c levels were associated with increased mean heart rate and reduced HRV, suggesting that chronic hyperglycemia directly contributes to autonomic impairment; importantly, the greatest HRV decline occurred within the first 5–10 years of diabetes duration (116). In a systematic review and meta-analysis, Benichou et al. similarly found that type 2 diabetes was associated with significant reductions in SDNN, RMSSD, pNN50, total power, LF, and HF, indicating impairment of both sympathetic and parasympathetic autonomic modulation (102). Prognostically, Cardoso et al. showed that reduced 24 h time-domain HRV parameters in patients with type 2 diabetes are associated with an approximately two-fold increased risk of cardiovascular events and all-cause mortality (117). Thus, diabetes mellitus, along with its duration and the degree of glycemic control, should be carefully considered when interpreting HRV measures.

### Heart failure

Heart failure is also characterized by autonomic imbalance, with sympathetic activation and reduced parasympathetic activity (118); in the UK-Heart study, lower SDNN strongly predicted mortality, with annual mortality rising from 5.5% when SDNN was >100 ms to 51.4% when SDNN was <50 ms (119). Similarly, Ponikowski et al. showed that depressed 24 h HRV independently predicted poor survival in moderate-to-severe chronic heart failure (120). In addition, a study by La Rovere et al. demonstrated that reduced short-term LF power during controlled breathing is a powerful and independent predictor of sudden cardiac death in patients with chronic heart failure (121). Thus, HRV reflects clinically relevant autonomic dysfunction and may help identify higher-risk heart failure patients. Moreover, heart failure patients are frequently treated with beta-blockers, ACE inhibitors, or ARBs, which can favorably modulate autonomic balance and improve HRV, and should therefore be considered when interpreting HRV measurements as discussed above.

### Metabolic syndrome and obesity

Metabolic syndrome is similarly linked to autonomic dysfunction, with meta-analytic evidence showing consistently lower 24 h SDNN, LF, VLF, and total power in patients with metabolic syndrome compared with controls, suggesting altered autonomic function (122). A study by Karason et al. demonstrated that obese individuals exhibit reduced SDNN, SDNN index, SDANN, and the square root of the power of LF and HF, all of which improve following weight loss (123). This reversibility is further supported by Jang et al., who showed that metabolic bariatric surgery significantly increased SDNN, RMSSD, LF power, and HF power, and reduced the LF/HF ratio, with postoperative weight loss independently associated with HRV improvement (124). Therefore, both metabolic syndrome and obesity significantly influence HRV by promoting autonomic imbalance, and their presence should be carefully considered when interpreting HRV measures.

### Ischemic heart disease

In ischemic heart disease, reduced HRV reflects impaired autonomic cardiovascular regulation, particularly diminished vagal modulation and/or relative sympathetic predominance. Huikuri and Mäkikallio emphasized that patients with ischemic heart disease show altered HRV compared with age-matched controls, with uncomplicated coronary artery disease mainly characterized by reduced HF oscillations, suggesting impaired vagal regulation, whereas prior myocardial infarction is associated with more global reductions in HRV. They further noted that reduced overall HRV is associated with increased mortality, nonfatal cardiac events, and life-threatening arrhythmias, especially after myocardial infarction (125). Similarly, a study by Wennerblom et al. showed that patients with uncomplicated coronary artery disease had significantly reduced global power, HF power, LF power, RMSSD, and pNN50 compared with healthy controls, supporting the concept that coronary artery disease itself can reduce HRV, mainly through reduced vagal tone (126). The prognostic importance of these findings was demonstrated by Kleiger et al., who found that among 808 post-acute myocardial infarction patients, a 24 h SDNN below 50 ms was associated with a 5.3-fold higher relative risk of mortality compared with a 24 h SDNN above 100 ms; importantly, reduced HRV remained an independent predictor of mortality after adjustment for clinical variables and ejection fraction (127). This association was reinforced by Buccelletti et al., whose meta-analysis showed that post-myocardial infarction patients with SDNN <70 ms on 24 h ECG recording had almost fourfold higher mortality risk over the subsequent three years (128).

### Hypertension

Hypertension is also associated with reduced HRV, highlighting the contribution of autonomic dysregulation to its pathophysiology and cardiovascular effects. In the ARIC study, Schroeder et al. evaluated 11,061 middle-aged participants and found that individuals with hypertension had lower HRV at baseline. Moreover, among participants without hypertension at baseline, lower HRV also predicted higher risk of incident hypertension over 9 years, suggesting that impaired autonomic regulation may precede and contribute to hypertension development (129). Similarly, the Ohasama study by Kikuya et al. showed that cardiovascular mortality increased with higher ambulatory blood pressure variability and lower daytime heart rate variability. Importantly, individuals with both elevated daytime systolic blood pressure variability and reduced daytime HRV had a particularly high risk of cardiovascular mortality, highlighting the prognostic significance of combined heart rate and blood pressure variability (130). Likewise, a study by Singh et al. showed that HRV was reduced in both men and women with systemic hypertension, supporting an association between hypertension and impaired autonomic regulation. Moreover, among normotensive men, lower baseline HRV (particularly lower LF power) predicted a higher risk of developing hypertension over 4 years, suggesting that autonomic dysregulation may be present early in the pathogenesis of hypertension (131).

### Atrial fibrillation

In atrial fibrillation (AF), HRV should not be interpreted as sinus-node variability, because the sinus node no longer determines the rhythm; rather, HRV reflects the irregular ventricular response produced by atrioventricular nodal filtering of rapid atrial impulses, with important autonomic modulation (132, 133). Van den Berg et al. showed that, despite the apparently “irregularly irregular” rhythm, HRV parameters in AF correlate with vagal tone, especially HF power, supporting the concept that HRV in AF can still carry autonomic information (133). This interpretation is clinically important because AF itself can produce higher HRV indices than sinus rhythm (133, 134); therefore, high HRV values in AF should not automatically be interpreted as “healthy” autonomic variability. Reduced HRV in chronic AF appears to identify a sicker phenotype, as lower HRV fraction was independently associated with older age, higher heart rate, depressed left ventricular ejection fraction, and diabetes (135). Prognostically, Yamada et al. demonstrated that reduced ventricular-response interval irregularity predicted cardiac mortality in chronic AF, independent of ejection fraction and ischemic AF (132). Thus, in AF patients, HRV is best interpreted as a marker of ventricular response complexity and autonomic/AV nodal modulation, with reduced irregularity potentially signaling impaired vagal modulation, adverse substrate, and higher cardiac mortality risk (132, 133).

Therefore, low HRV should not be interpreted as an isolated marker of “poor autonomic tone” without considering the patient's comorbidity profile; rather, it may represent both disease-related autonomic remodeling and a prognostic signal identifying patients at higher cardiovascular and mortality risk. Moreover, patients with chronic diseases are more likely to be receiving medications that may influence HRV, as discussed above.

Figure 1 illustrates a stepwise framework for the context-aware interpretation of heart rate variability.

**Figure 1:**
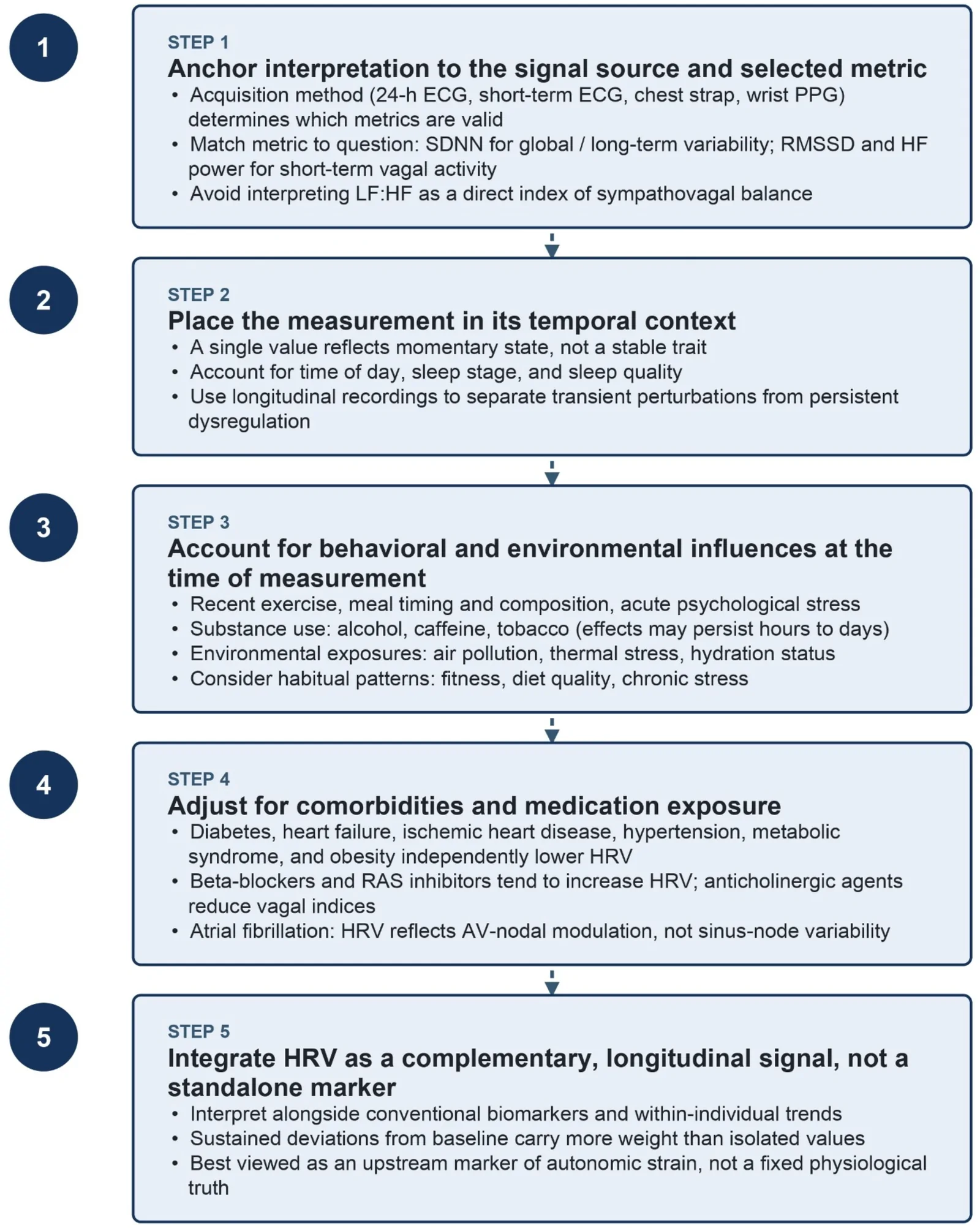
A stepwise framework for context-aware interpretation of heart rate variability. HRV, heart rate variability; ECG, electrocardiogram; PPG, photoplethysmography; SDNN, standard deviation of All N-N intervals; RMSSD, root mean square of successive differences; HF, high-frequency; LF, low-frequency; RAS, renin–angiotensin system.

## Conclusion

HRV is a promising, non-invasive marker of autonomic regulation and cardiovascular risk, but its interpretation requires careful attention to context. Because HRV is influenced by measurement modality, recording duration, circadian timing, lifestyle factors, medications, and comorbidities, isolated values should not be interpreted as direct indicators of autonomic health. A context-aware approach may improve the clinical utility of HRV and support its role as a complementary tool for cardiovascular risk assessment.
